# Clinical utility of synuclein skin biopsy in the diagnosis and evaluation of synucleinopathies

**DOI:** 10.3389/fneur.2024.1510796

**Published:** 2024-12-18

**Authors:** Jonathan R. Isaacson, Roy Freeman, Christopher H. Gibbons

**Affiliations:** Center of Autonomic and Peripheral Nerve Disorders, Beth Israel Deaconess Medical Center, Boston, MA, United States

**Keywords:** Parkinson’s disease, alpha-synuclein, skin biopsy, synucleinopathy, REM sleep behavioral disorder, multiple system atrophy, Lewy body dementia

## Abstract

**Introduction:**

The diagnosis of diseases known as synucleinopathies, Parkinson’s disease (PD), multiple system atrophy (MSA) and Lewy body dementia (DLB), is predominantly based on clinical criteria. However, diagnostic uncertainty may persist until late in the disease process leading to delays in diagnosis and medical mismanagement. Skin biopsy detection of phosphorylated alpha-synuclein (P-SYN) is a sensitive and specific technique that increases diagnostic sensitivity of synucleinopathies, although the clinical utility of this test has not been fully explored.

**Methods:**

To determine the role of skin biopsy in the diagnosis of synucleinopathies we performed a retrospective chart review of patients who underwent skin biopsy for detection of P-SYN in the evaluation of neurodegenerative disease at a tertiary care academic institution to investigate the change in diagnosis and medical management based on the results of skin biopsy detection of P-SYN.

**Results:**

We included 97 patients suspected to have a synucleinopathy: 54 with PD, 19 with DLB and 24 with MSA. After skin biopsy testing for P-SYN, 78% of patients had a change in their clinical care with 66% having a change in their diagnosis and 55% having a change in their treatment. Changes in diagnosis were most common in patients with parkinsonism with prominent action tremor (93%), lower-extremity predominant parkinsonism (postural instability and gait dysfunction) (90%), and parkinsonism with predominant cognitive dysfunction (76%).

**Discussion:**

In patients with suspected synucleinopathies, skin biopsy detection of P-SYN had a high level of clinical utility leading to changes in clinical diagnosis and treatment.

## 1 Introduction

The diagnosis of disorders characterized as ‘synucleinopathies’ is primarily based on clinical phenomenology that includes motor, autonomic and/or cognitive features. The central synucleinopathies, Parkinson’s disease (PD), multiple system atrophy (MSA), and Lewy body dementia (DLB), have features that overlap with one another and with other neurodegenerative diseases such as Alzheimer disease, and the tauopathies, corticobasal degeneration (CBD) and progressive supranuclear palsy (PSP). Other non-degenerative diseases such a cerebrovascular disease, drug-induced parkinsonism, gait disorders, and essential tremor are also commonly in the differential diagnosis. Diagnostic uncertainty may persist until late in the disease process when substantial neurodegeneration has occurred.

Diagnostic errors or uncertainty can lead to inappropriate treatment, increased risk of adverse events, challenges to life planning, and delayed or inappropriate enrollment in investigational trials (1). There is an unmet need for an early and accurate diagnostic biomarker for these disorders to improve patient care and clinical outcomes.

In an effort to advance the diagnostic algorithm of the Lewy body diseases by linking clinical phenomenology with pathology, recently proposed updates to the classification schemes have included alpha-synuclein biomarkers. Within this context, skin biopsy detection of phosphorylated alpha-synuclein (P-SYN) has emerged as an accessible pathologic biomarker for parkinsonian syndromes (2–9). However, despite recent publications reporting the high sensitivity and specificity of skin biopsy in patients with synucleinopathies (10, 11), the utility of this test in clinical practice has not been fully explored. The goal of this study is to examine the role of skin biopsy in the evaluation and management of patients with a suspected synucleinopathy of uncertain etiology seen at a tertiary referral center.

## 2 Patients and methods

### 2.1 Patient population

A retrospective chart review was performed on all patients who underwent skin biopsy for detection of P-SYN in the evaluation of neurodegenerative disease at our institution between Jan 2021 and Dec 2023. The study was approved by the Beth Israel Deaconess Institutional Review Board. Patients with suspected synucleinopathy were referred for skin biopsy by their treating neurologist because of diagnostic uncertainty. Demographic information, clinical history, physical examination, skin biopsy results, treatment decisions and changes to diagnosis were obtained by chart review.

Clinical diagnosis prior to the skin biopsy result was defined by primary ICD-10 codes used at the time of the skin biopsy. If ICD-10 codes were not available in the chart, the primary diagnosis reported by the treating clinician was included. Clinical diagnosis after the skin biopsy was defined as the ICD10 code (or primary diagnosis by treating physician if not available) at the clinical visit after obtaining skin biopsy results. Physician specialty and reason for referral were captured. Changes to treatment were defined as stopping, modifying, or initiating a medication based on the skin biopsy results. Physician and patient responses to abnormal or normal test results were captured when available in the medical record and included changes in treatment, diagnosis, referrals, testing or prognosis. Patients were excluded if clinical data were not available either before or after biopsy results, or if the clinical data suggested a diagnosis of pure autonomic failure without evidence of a central neurodegenerative disease process.

### 2.2 Skin biopsy procedure

Three-millimeter punch biopsies were obtained from the leg 10 cm above the lateral malleolus, 10 cm above the lateral knee, and 2 cm lateral to posterior cervical C7 prominence to the depth of the subcutaneous fat using standard techniques and locations (12). Skin biopsy specimens were fixed in Zamboni paraformaldehyde solution in pre-labeled tubes and shipped overnight for processing (CND Life Sciences, Scottsdale AZ). At the central processing site, biopsies were washed and placed in a 20% glycerol cryoprotectant solution. The biopsies were then sectioned into 50 micron thickness and dual immunostained by antibodies to protein gene product 9.5 (PGP 9.5), a pan-axonal marker, and phosphorylated alpha-synuclein (P-SYN) as previously reported (13). The biopsies were viewed by confocal microscopy to identify nerve fibers stained by PGP 9.5 and by P-SYN and the merged image was used to confirm the presence of intra-axonal P-SYN.

### 2.3 Statistical analysis

Statistical analysis was performed using SPSS v17.0 (IBM, Chicago, IL). Data are presented using descriptive statistics with group data presented as mean ± standard deviation. Skin biopsy results are reported as binary outcomes (present or absent P-SYN). Tabular data are presented as number of cases in each group, with chi-square or contingency table analysis of results. Differences between groups were analyzed by ANOVA with the Holm–Sidak *post hoc* test when normally distributed (Shapiro–Wilk). If data were not normally distributed, Kruskal-Wallis with Dunn’s method for post-hoc analysis was used.

## 3 Results

**Figure 1:**
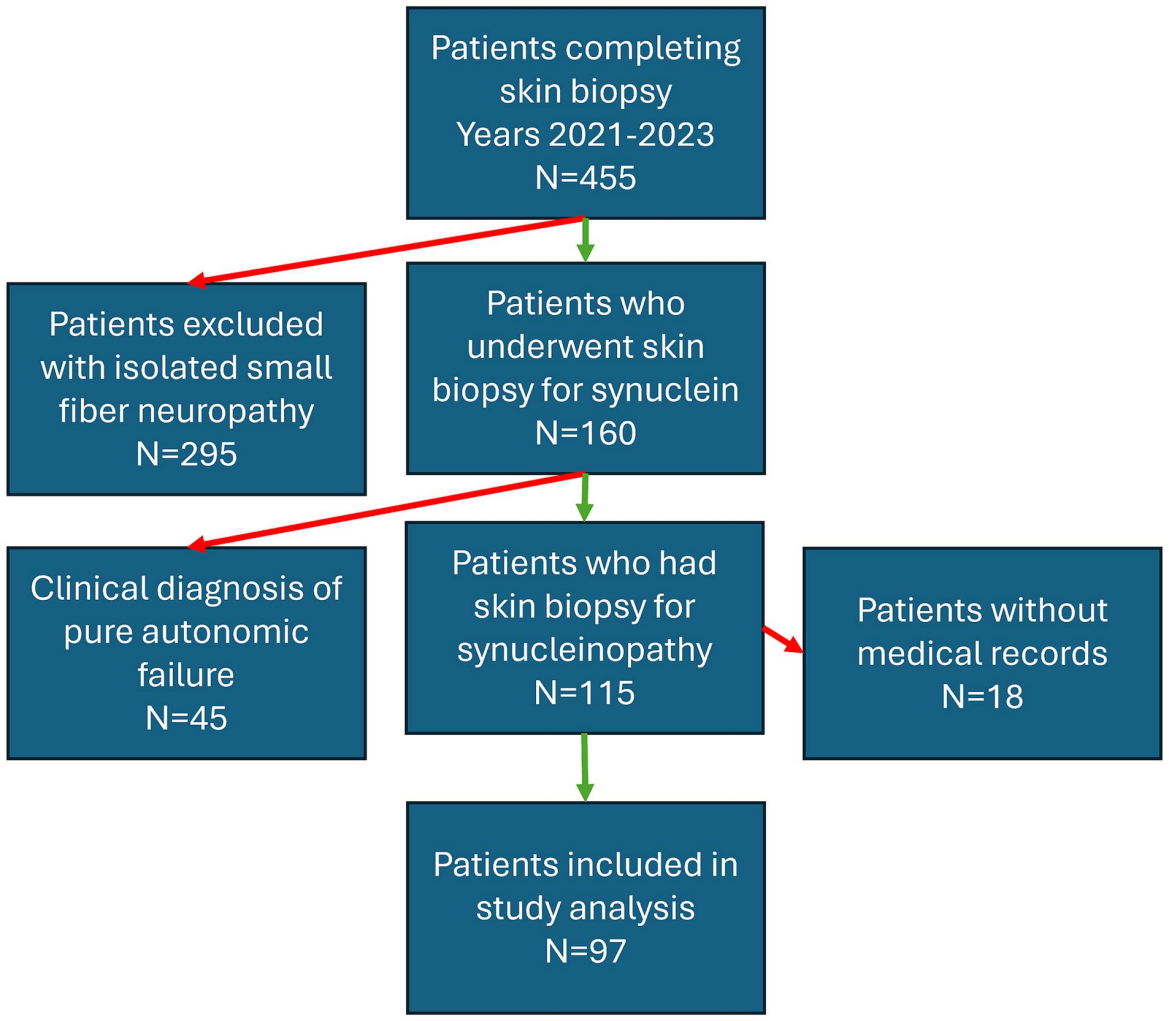
Flowchart depicting the inclusion criteria for patients who underwent skin biopsy at Beth Israel Deaconess Medical Center over a three-year period (2021–2023) for the indication of parkinsonism. Red arrows indicate excluded groups, green arrows indicate included patients.

Ninety-seven consecutive patients who completed skin biopsies for the evaluation of a synucleinopathy were included in the study (mean age 71 years, range 52–89) (Figure 1 and Table 1). Patients were referred to our center by movement disorder specialists (75%), general neurologists (7%), and cognitive disorder specialists (18%). The most common reasons for referral are outlined in Table 1. Overall, P-SYN was detected in 63 of 97 (65%) referred patients. In this cohort, skin biopsies were more likely to contain P-SYN in patients with parkinsonism with prominent autonomic features, parkinsonism with prominent action tremor, and parkinsonism with inadequate levodopa response (all groups with >70% P-SYN positivity) and least likely to contain P-SYN in suspected drug-induced parkinsonism (11%) (see Table 1).

**Table 1:** Demographic information and skin biopsy findings grouped by clinical presentation.

Clinical presentation	Age (mean ± SD)	*N* (%)	Sex (M/F)	Skin biopsy with P-SYN	Normal skin biopsy
Postural instability and gait dysfunction	71 ± 9	21 (22%)	17/4	14 (67%)	7 (33%)
Parkinsonism with prominent autonomic features	71 ± 8	21 (22%)	16/5	18 (86%)	3 (14%)
Suspected synucleinopathy with cognitive dysfunction	72 ± 10	21 (22%)	10/11	13 (62%)	8 (38%)
Prominent action tremor	71 ± 7	15 (15%)	13/2	11 (73%)	4 (27%)
Parkinsonism with inadequate levodopa response	71 ± 8	14 (14%)	12/2	10 (71%)	4 (29%)
Suspected drug-induced parkinsonism	71 ± 9	9 (9%)	4/5	1 (11%)	8 (89%)

Table provides an overview of both the findings from skin biopsies and their impact on diagnosis and treatment changes across various clinical scenarios of indeterminate parkinsonism.

### 3.1 Impact on diagnosis and treatment

Skin Biopsy results (both positive and negative) led to changes in diagnosis in 66% of patients, changes in treatment in 55% of patients, and changes in both diagnosis and treatment in 41% of patients (see Figure 1). In total, 78% of patients (76 of 97) had a change in diagnosis and/or treatment after receipt of the skin biopsy results.

### 3.2 Change in diagnosis

A skin biopsy was likely to result in a diagnosis change in both P-SYN positive and negative patients [60% in P-SYN positive (38 of 63) and 76% (26 of 34) in P-SYN negative patients]. Changes in diagnosis were most common in patients referred with parkinsonism with prominent action tremor (93%), lower-extremity predominant parkinsonism (postural instability and gait dysfunction) (90%), and parkinsonism with predominant cognitive dysfunction (76%).

**Figure 2:**
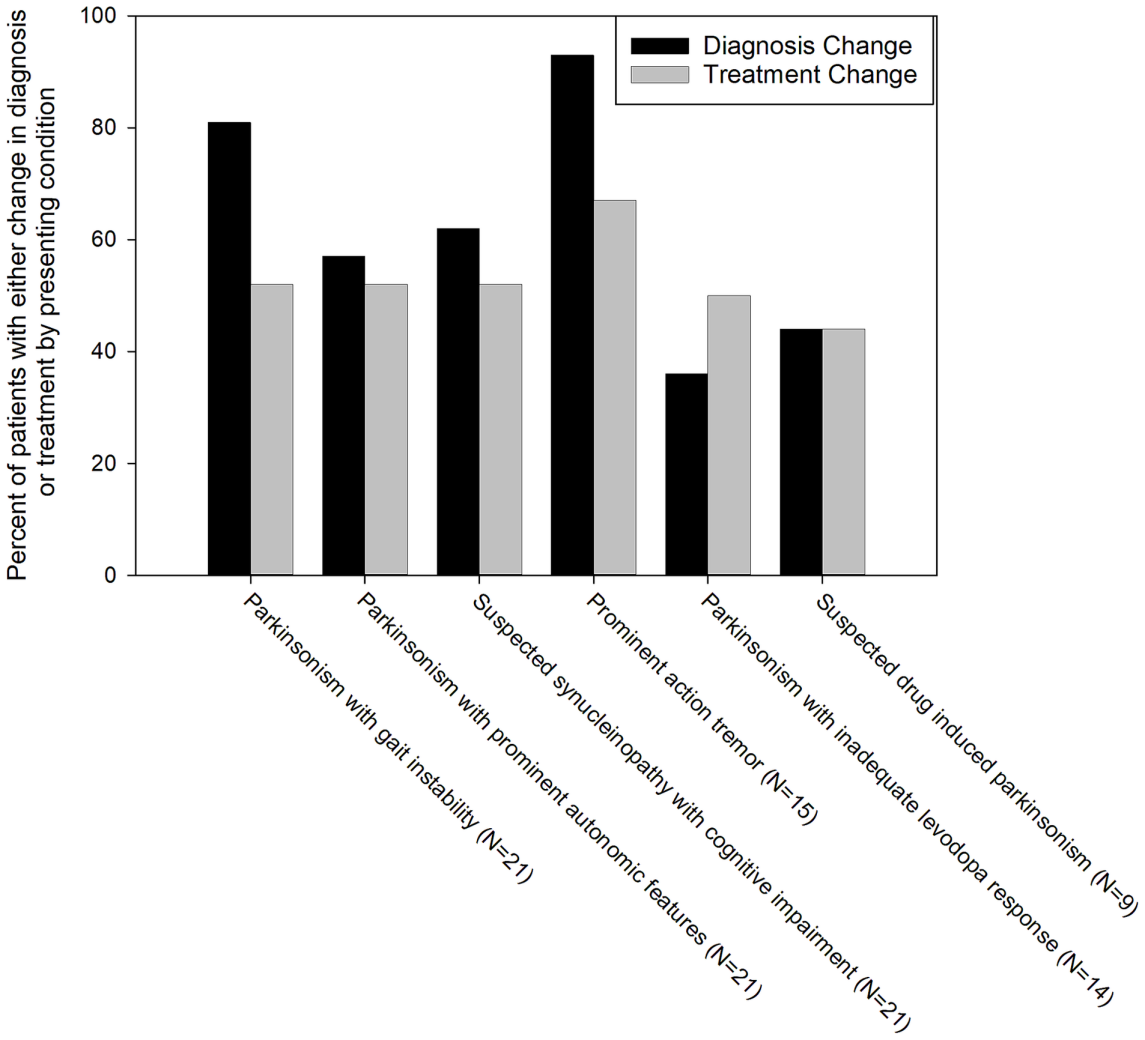
Percentage of patients with a change in diagnosis and treatment grouped by reason for referral for skin biopsy. Black bars represent a change in diagnosis, grey bars represent a change in treatment.

Patients in whom skin biopsy was performed to evaluate a prominent action tremor had a differential diagnosis that included essential tremor and the tremors of Parkinson’s disease. The skin biopsy was positive in 73% (11/15) in patients with prominent action-tremor, with a change in diagnosis in 91% (10 of 11) of the positive cases, and 100% (4 of 4) of the P-SYN negative cases (Figure 2). Patients in whom skin biopsy was performed to evaluate parkinsonism with prominent autonomic features had a differential diagnosis that included multiple system atrophy and Parkinson’s disease. The skin biopsy was positive in 86% (18 of 21), with a change in diagnosis in 50% (9 of 18) of positive cases, and 100% (3 of 3) of the negative cases. Patients in whom a skin biopsy was performed to evaluate parkinsonism with prominent cognitive dysfunction had a differential diagnosis that included Parkinson’s disease, Lewy Body dementia, Alzheimer’s disease, normal pressure hydrocephalus and pseudodementia. Skin biopsy was positive in 62% of cases (13 of 21), with a change in diagnosis in 46% (6/13) of positive cases, and 88% (7/8) of negative cases. Patients in whom a skin biopsy was performed to evaluate lower extremity predominant parkinsonism [postural instability and gait dysfunction (PIGD)] had a differential diagnosis that included Parkinson’s disease, cerebrovascular disease, musculoskeletal disease, and functional neurological disorder. Skin biopsy was positive in 67% of cases (14 of 21), with a change in diagnosis in 86% of cases (12 of 14) in positive cases and 100% (7 of 7) of negative cases. For patients in whom skin biopsy was performed to evaluate unclear levodopa response, the differential diagnosis included Parkinson’s disease, multiple system atrophy and progressive supranuclear palsy. Skin biopsy was positive in 71% of cases (10 of 14), with a change in diagnosis in 80% (8 of 10) of the positive cases, and 75% (3 of 4) of negative cases. For patients in whom a skin biopsy was performed to evaluate suspected drug-induced parkinsonism the differential diagnosis included drug-induced parkinsonism or Parkinson’s disease. Skin biopsy was positive in 11% (1 of 9) of cases, with a change in diagnosis in 100% (1 of 1) in positive cases, and 38% (3 of 8) of negative cases.

### 3.3 Change in treatment

**Figure 3:**
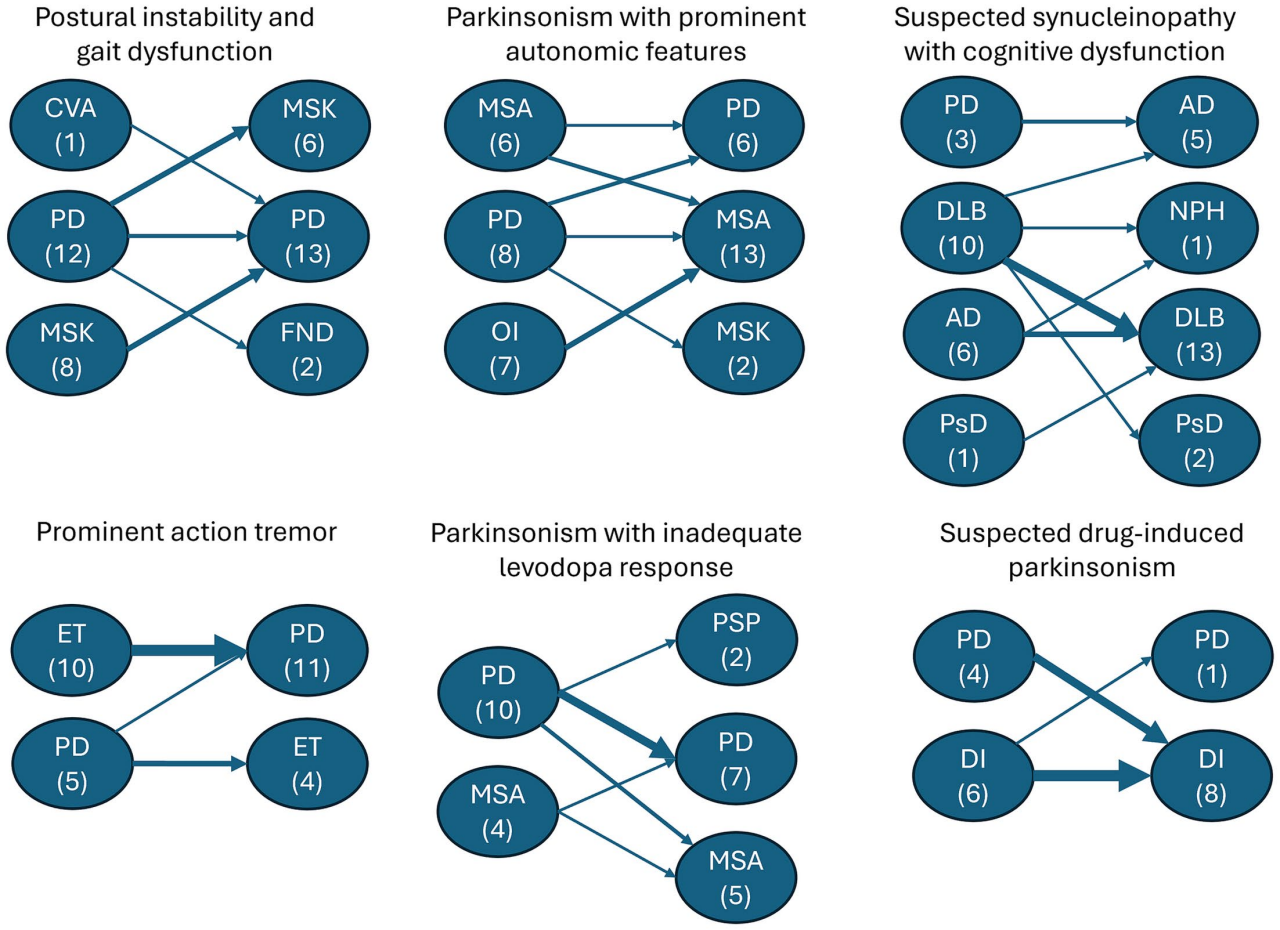
Flowchart depicting the clinicians’ pre-biopsy diagnosis grouped by the categories from Table 1 that include (1) postural instability and gait dysfunction, (2) parkinsonism with prominent autonomic features, (3) suspected synucleinopathy with cognitive dysfunction, (4) prominent action tremor, (5) parkinsonism with inadequate levodopa response and (6) suspected drug induced parkinsonism. Arrows indicate the shift from the pre-biopsy diagnosis to the post-biopsy diagnosis. Arrow thickness represents the outcome frequency. AD, Alzheimer’s Disease; CVA: Cerebrovascular accident; DI, drug-induced parkinsonism; DLB, dementia with Lewy bodies; ET, essential tremor; FND, functional neurological disorder; MSA, multiple system atrophy; MSK, musculoskeletal; NPH, normal pressure hydrocephalus; OI, orthostatic intolerance; PD, Parkinson’s disease; PsD, pseudodementia; PSP, progressive supranuclear palsy.

Skin biopsy led to a change in treatment in 55% of patients. Treatment changes were highest in patients with parkinsonism and prominent action tremor (67%) and lowest in suspected drug-induced parkinsonism patients (44%) (Figure 3). In patients presenting with parkinsonism and a prominent action tremor, skin biopsy was positive for P-SYN 73% of the time, and a treatment change was made 67% of the time. In patients with parkinsonism and an unclear levodopa response, skin biopsy was positive 71% of the time, and a treatment change was made in 50% of the cases. In patients presenting with parkinsonism and prominent cognitive dysfunction, skin biopsy was positive in 62% of cases, and a treatment change was made in 57% of the cases. In patients with gait dysfunction, skin biopsy was positive in 67% of the cases, and a treatment change was made in 45% of the cases. In patients with suspected drug-induced parkinsonism, skin biopsy was positive in 11% of the cases, and a treatment change was made in 44% of the cases. In patients with Parkinsonism with prominent autonomic features, skin biopsy was positive in 86% of the cases, and a treatment change was made in 52% of the cases. In total, 78% of patients (76 of 97) had a change in diagnosis and/or treatment after receipt of the skin biopsy results. The number of patients in the various diagnostic categories before and after skin biopsy is detailed in Figure 3.

After biopsy results, treatment changes were as follows:

1. Levodopa was started in 25 patients with post-biopsy synucleinopathy diagnoses.
2. Levodopa was discontinued in 16 patients with post-biopsy diagnoses that included progressive supranuclear palsy, drug-induced parkinsonism, essential tremor and essential tremor-plus, normal pressure hydrocephalus, functional neurological disorder, and pseudodementia.
3. Medications for essential tremor were started in 3 patients with post-biopsy essential tremor diagnoses.
4. Medications for esstential tremor were discontinued in 2 patients with P-SYN on biopsy.
5. Antidepressant medications were started in 4 patients without P-SYN on biopsy.
6. Dopamine antagonists were discontinued in 10 patients with diagnoses that included drug-induced parkinsonism and Lewy body dementia.
7. Treatment for orthostatic hypotension was started in 6 patients.
8. After biopsy results, 8 new referrals were made to psychiatry, physical and occupational therapy, orthopedic surgery for spine evaluation, and neurosurgery for shunt evaluation.
9. In 24 patients, skin biopsy results led to family meetings to discuss diagnoses, prognoses, and life-planning.

## 4 Discussion

Features inherent to the clinical utility of a test are that it provides accurate, reliable, and actionable information to guide appropriate treatment decisions that improve clinical outcomes and optimize health care utilization (14). In this study, we build on the evidence-base showing the accuracy and reliability of skin biopsy detection of alpha-synuclein to show how test results are used to inform clinical decision making in a tertiary care academic clinical neurology practice.

The clinical questions investigated by the skin biopsy encompassed a range of movement, cognitive and autonomic disorders. We report that skin biopsy (1) changed the underlying diagnosis in 65% of patients, (2) changed the underlying treatment in 55% of patients, and that (3) changes to diagnosis or treatment were independent of the specific skin biopsy results, i.e., whether biopsy was positive or negative.

Skin biopsy detection of P-SYN has emerged as a minimally invasive test with high sensitivity and specificity in accurately diagnosing synucleinopathies (8–10, 12, 15–18). While data on sensitivity and specificity have been reproduced in many large studies from different academic centers, there are limited data on the clinical utility of skin biopsy in patients with suspected synucleinopathies with diagnostic uncertainty. In addition, recent studies have demonstrated the potential to differentiate among synucleinopathy subtypes (such as MSA from PD) based on synuclein deposition and regional topography, although it is unclear if this has been incorporated into clinical decision making (19–21). There is a significant need for understanding the utility of this test across the range of synucleinopathies, particularly those considered clinically indeterminate, but even in those in which the diagnosis is more certain.

Although the clinical diagnostic accuracy of Parkinson’s disease has improved over the past two decades accurate diagnosis remains challenging, particularly in in the hands of non-expert practitioners and in the early disease stages (22–24). Similarly, the accurate diagnosis of multiple system atrophy has improved, but the diagnosis continues to be challenging (25, 26). Early and accurate diagnosis has both clinical and research implications. Clinically, diagnostic delays and misdiagnosis may increase risks to morbidity and mortality through medication mismanagement and unrealistic expectations on outcomes. For research studies, delays and misdiagnoses can preclude patients from enrolling in clinical trials or result in greater study heterogeneity with an accompanied greater risk of study failure.

The empirical clinical subtypes of PD, tremor-predominant Parkinson’s disease, postural instability and gait dysfunction (27) and their associated non-motor features, cognitive impairment and autonomic dysfunction (28) provide a framework for understanding the diagnostic challenge faced by clinician caring for patients with parkinsonian features. While reports suggest these subtypes may not be stable over time (29), at the time of presentation they impose diagnostic challenges with several different differential diagnoses each with important clinical implications. The reasons for biopsy in this study (see Table 1) reflect those challenges.

### 4.1 Postural instability and gait dysfunction

Gait dysfunction was among the most common reasons for ordering a skin biopsy in this cohort. The differential diagnosis of patients with this presentation included the postural instability and gait dysfunction subtype of Parkinson’s disease, vascular parkinsonism, multiple system atrophy, the tauopathies (progressive supranuclear palsy and corticobasal syndrome) and musculoskeletal disorders such as osteoarthritis of the spine and lower extremity joints (30). The response to therapy, natural history, and prognosis vary widely among these disorders underscoring the importance of accurate diagnosis. In the present study, clinical diagnosis and treatment changes were frequent after biopsy (Figures 2, 3). In this group, all patients with an initial working diagnosis of musculoskeletal disease had a follow up diagnosis of Parkinson’s disease, half of the patients with an initial diagnosis of PD had a follow up diagnosis of musculoskeletal disease, and no patients had a follow up diagnosis of cerebrovascular disease. Notably, 26% patients had levodopa initiated and 17% of patients had levodopa discontinued after biopsy results.

### 4.2 Autonomic dysfunction

Autonomic dysfunction is a common associated feature of Parkinson’s disease, particularly late in the clinical course, and is one of the clinical hallmarks of multiple system atrophy. In contrast, clinically meaningful autonomic dysfunction rarely occurs in the tauopathies, PSP and CBD. Autopsy studies emphasize the diagnostic challenges that parkinsonian patients with autonomic dysfunction impose; they are frequently misdiagnosed as MSA (21). In the present study, almost 90% of the patients with prominent autonomic dysfunction had positive biopsies which led to changes in diagnosis and treatment in over 50% of patients. Two with PD were re-diagnosed as MSA and two MSA patients, were re-diagnosed as PD. The biopsies from patients with MSA were noted to have greater deposition of P-SYN at all 3 biopsy locations and to have more distal somatic P-SYN deposition within regions of the subepidermal plexus (19). The patients re-diagnosed as PD were noted to have lower total amounts of P-SYN deposition that was primarily within the posterior cervical biopsy and involved autonomic nerve fibers; and P-SYN was not in the distal leg or distal thigh biopsies (19, 21), there was no reported P-SYN within the subepidermal plexus of those patients re-diagnosed as PD. The reported differences in the amount and distribution of alpha-synuclein deposition across the 3 biopsies, and within the different nerve fiber subtypes presumably played a role in the reclassification (19, 21). Although a recent study has reported the presence of P-SYN within Schwann cells as a sensitive and specific marker for patients with MSA, this technique was not employed in the routine analysis of these skin biopsies for P-SYN (31).

### 4.3 Cognitive impairment

Cognitive impairment was a frequent cause for biopsy in this study. These findings are consistent with the evidence that cognitive impairment is a frequent non-motor manifestation of PD and may manifest throughout the clinical course. Dementia occurs in up to 90% of patients with Parkinson’s disease after 25 years (32) and is present in approximately 20% of patients with newly diagnosed PD, when the diagnosis is most challenging (33).

Cognitive impairment precedes the motor features of PD and may be present in individuals at risk for Parkinson’s disease, e.g., those with anosmia (34). The differential diagnosis in this population includes dementia with Lewy bodies, and Alzheimer disease. Differentiation between Lewy body diseases and AD is now of increasing importance given the differences in the clinical course, mortality rate, survival times and the availability of disease modifying therapies for AD (35). These factors appeared to underlie the use of skin biopsy in the parkinsonian patients with cognitive impairment or patients with cognitive impairment and a suspected synucleinopathy. Although skin biopsy alone cannot differentiate between PD and DLB, a recent report suggests that greater P-SYN deposition is seen in DLB compared to PD, and that more widespread denervation of small unmyelinated C-fibers is often linked to DLB (10). It appears that the combination of the pathologic features (P-SYN amount and location) with the clinical phenotype helped to refine the clinical diagnosis – a diagnosis change occurred in 62% percent of these patients, and a change in treatment occurred in 58% of these patients. For example, the recognized neuroleptic sensitivity of patients with Lewy body dementia led to the discontinuation of these agents in 4 patients with a post-biopsy diagnosis of Lewy body dementia based on widespread P-SYN deposition within autonomic nerve fibers.

Finally, the coexistence of multiple pathologies, e.g., Alzheimer and Lewy body pathology is now well recognized to play a role in the clinical phenotype, clinical course, prognosis and possibly in the response to therapy of patients with Alzheimer disease. Recognition of the presence of synuclein may assist in the management of these patients (36, 37).

### 4.4 Tremor

Tremor was among the most common reasons for carrying out a skin biopsy in parkinsonian patients. Tremor is present in up to 95% of PD patients and has several clinical presentations (38). Resting tremor is the most common tremor, but other tremor types such as postural tremor [with variants such as re-emergent tremor (39) and kinetic tremors] occur in a substantial number of patients (38). Many patients manifest more than one tremor type (40).

The most important differential diagnosis for the tremors of PD is essential tremor, a highly prevalent disorder present in approximately 1% of the population (41). A small, but clinically important, percentage of essential tremor patients phenoconvert to PD (42). Essential tremor may have additional clinical features such as dystonia, subtle parkinsonian signs or memory loss; a disorder called essential tremor-plus by some investigators. It is not known whether these patients have an increased likelihood of phenoconversion to PD. These diagnostic challenges, in part, appeared to drive the use of skin biopsy, which together with the clinical course led to diagnostic changes in 93% and treatment changes (initiation or discontinuation of essential tremor treatment) in 67% of patients (see Figures 2, 3).

### 4.5 Levodopa response

Multiple system atrophy is characterized by a limited and poorly sustained response to levodopa and dopamine agonists – a substantial and persistent response to dopaminergic agents is an exclusion criterion for clinically established and clinically probable MSA in the most recent MDS diagnostic criteria (43). PSP and CBD are similarly poorly responsive to dopaminergic agents. In this study, based on skin biopsy results 11% of PD patients were reclassified as PSP and 7% as MSA.

### 4.6 Drug-induced parkinsonism

Drug-induced parkinsonism is among the most common causes of parkinsonism that is not due to neurodegeneration. The prevalence, in some studies reaches approximately one third of that of idiopathic PD (44, 45). Accurate diagnosis has important implications for prognosis and treatment, which in drug-induced parkinsonism includes the discontinuation of dopamine receptor blocking drugs. In this study 89% of patients in whom drug-induced parkinsonism was considered in the differential diagnosis had a negative biopsy. The diagnosis changed from idiopathic PD to drug-induced parkinsonism in 45% of patients with subsequent discontinuation of dopamine antagonists.

## 5 Conclusion

The results of this study underscore the importance of the role of this neurodiagnostic test in facilitating referrals to non-neurological medical specialties and rehabilitation disciplines such as physical, occupational and speech therapy. Of equal importance, is the value of counseling and accurate prognostication to assist with life-planning, particularly in the absence of effective disease modifying therapy. Family meetings were held to discuss these issues in over 25% of patients after biopsy results were obtained.

## 6 Limitations

Our study had several limitations. These include the retrospective nature of the study and the small sample size at a single medical center. Further, chart reviews often require interpretation of clinical results that could introduce outcome bias. Although we had strict prespecified criteria to define changes in diagnosis and treatment, this approach is not as robust as a prospective, blinded outcome study. Accurate clinical diagnosis is an iterative process, requiring frequent clinical assessments and diagnostic tests including skin biopsy, biofluid diagnostic tests, and imaging. In this study, we could not determine the relative contribution of other components of the diagnostic process. An important facet of clinical utility is the impact that positive or negative test results have for patients and physicians beyond diagnosis and clinical decision making. The doctor-patient relationship and other social and emotional factors are topics for future study. Lastly, the study site is a large academic center, so the patients reported may not be generalizable to other populations. Despite these limitations, skin biopsy detection of P-SYN appears to have high clinical utility in patients with suspected synucleinopathy and diagnostic uncertainty.

## Data Availability

The raw data supporting the conclusions of this article will be made available by the authors, without undue reservation.
